# Musculoskeletal Manifestations of T-Cell Lymphomas: Clinical Presentation, Imaging Features, Pathobiology, and Orthopedic Considerations—A Narrative Review

**DOI:** 10.3390/cells15151332

**Published:** 2026-07-25

**Authors:** Ibrahim Alshaygy, Abdulaziz S. AlNahari, Mohannad W. Awwad, Badr Fadhel Alshehri, Hayfaa Saud Alshaalan, Waleed Albishi, Motaz Alaqeel, Abdulrahman Alaseem

**Affiliations:** 1 Department of Orthopedic Surgery, College of Medicine, King Saud University, Riyadh 11461, Saudi Arabia; 2 College of Medicine, King Saud University, Riyadh 11461, Saudi Arabia

**Keywords:** T-cell lymphoma, musculoskeletal lymphoma, bone lymphoma, extranodal lymphoma, pathological fracture, tumor microenvironment, orthopedic oncology, soft tissue mass, peripheral T-cell lymphoma, lymphoma imaging

## Abstract

Lymphomas are a heterogeneous group of aggressive lymphoid malignancies characterized by frequent extranodal involvement. Musculoskeletal manifestations, although uncommon, represent a clinically significant and under-recognized presentation that often mimics primary bone tumors, soft tissue sarcomas, and infectious conditions, leading to diagnostic delays and inappropriate initial management. This narrative review synthesizes current evidence on the clinical presentation, imaging characteristics, pathology, and biological basis of musculoskeletal T-cell lymphomas, with particular emphasis on implications for orthopedic practice. A structured literature search was conducted across major databases from 2000 to the present, and evidence was integrated across clinical, radiologic, pathologic, and therapeutic domains. Musculoskeletal involvement most commonly presents with localized bone pain, soft tissue masses, or pathological fractures, with imaging features such as diffuse marrow infiltration, soft tissue extension, and relatively preserved cortical bone serving as important diagnostic clues. Definitive diagnosis relies on tissue biopsy with comprehensive immunophenotypic and molecular characterization. Emerging insights into the tumor microenvironment highlight its role in disease progression and therapeutic response, offering potential avenues for targeted treatment strategies. Management is subtype-specific and centers on systemic therapy, with selective roles for radiotherapy and orthopedic intervention in cases of structural compromise. Early recognition through integrated clinical, imaging, and pathological assessment, combined with multidisciplinary collaboration, is essential to optimize outcomes in this rare but challenging disease entity.

## 1. Introduction

T-cell lymphomas, collectively classified as mature T- and NK-cell neoplasms, represent a heterogeneous group of post-thymic lymphoid malignancies comprising more than 30 distinct subtypes according to the current 5th edition World Health Organization (WHO-HAEM5) and 2022 International Consensus Classification (ICC) systems [1]. These entities are broadly categorized based on clinical presentation and cellular origin into nodal and extranodal forms. Among the most clinically relevant subtypes are peripheral T-cell lymphoma, not otherwise specified (PTCL-NOS)—a diagnosis of exclusion and the most common subtype in Western populations—anaplastic large-cell lymphoma (ALCL), further divided into ALK-positive (associated with ALK gene fusions and relatively favorable prognosis) and ALK-negative variants (molecularly heterogeneous with variable outcomes), extranodal NK/T-cell lymphoma (ENKTCL), an Epstein–Barr virus (EBV)-driven aggressive lymphoma with predilection for the nasal cavity and upper aerodigestive tract, and adult T-cell leukemia/lymphoma (ATLL), a human T-lymphotropic virus type 1 (HTLV-1)-associated malignancy endemic to specific geographic regions including southwestern Japan, the Caribbean, and intertropical Africa [2]. Overall, T-cell lymphomas account for approximately 10–15% of non-Hodgkin lymphomas in Western populations and up to 20% in Asia, with ENKTCL being more prevalent in East Asia and ATLL confined to HTLV-1-endemic regions. Despite advances in classification and therapy, prognosis remains poor across most subtypes, with a 5-year overall survival of approximately 30%, which is inferior to that observed in B-cell lymphomas treated with comparable regimens, with the notable exception of ALK-positive ALCL [3].

Extranodal involvement is a defining feature of T-cell lymphomas and carries important prognostic implications. In the landmark International Peripheral T-Cell Lymphoma Project (IPTCLP), 62% of patients with PTCL-NOS exhibited extranodal disease at diagnosis, including 49% with combined nodal and extranodal involvement and 13% with exclusively extranodal presentation; commonly affected sites included the skin, bone marrow, liver, gastrointestinal tract, and lung, and extranodal disease was identified as an independent adverse prognostic factor [4]. Within this spectrum, musculoskeletal involvement—including bone, bone marrow, and skeletal muscle—represents an uncommon yet clinically significant manifestation. Secondary bone involvement has been reported in approximately 15% of disseminated lymphomas, whereas skeletal muscle involvement, particularly primary skeletal muscle PTCL-NOS, remains exceedingly rare and is largely described in isolated case reports. Such presentations often manifest as soft tissue masses or diffuse muscle enlargement, mimicking inflammatory myopathies or soft tissue sarcomas, thereby posing substantial diagnostic challenges and frequently portending an aggressive disease course [5].

When T-cell lymphomas involve the musculoskeletal system, their clinical and radiological features frequently overlap with common orthopedic and infectious conditions. Presentations such as localized bone pain, soft tissue swelling, pathological fractures, and elevated inflammatory markers may closely resemble osteomyelitis, septic arthritis, or primary bone tumors, often leading to misdiagnosis and prolonged empirical treatments. In a representative case series, bone involvement by lymphoma was observed in 16–20% of patients; however, non-specific imaging findings and overlapping laboratory features frequently resulted in initial referral to orthopedic or infectious disease services rather than hematologic evaluation [6]. The consequences of such diagnostic delays can be substantial. In a retrospective cohort of 104 lymphoma patients, delays exceeding six months from symptom onset to definitive diagnosis were associated with significantly worse overall survival (44% vs. 72% in patients diagnosed earlier; *p* = 0.009), highlighting that while short delays may be unavoidable, prolonged diagnostic uncertainty—particularly in aggressive lymphomas presenting with atypical musculoskeletal features—can lead to disease progression, advanced stage at presentation, and inferior clinical outcomes [7].

In this context, the present narrative review aims to synthesize current evidence on the musculoskeletal manifestations of T-cell lymphomas, with particular emphasis on clinical presentation and diagnostic challenges. In addition, it provides a comprehensive overview of the underlying pathology, immunophenotype, and biological mechanisms, including the role of the tumor microenvironment. Finally, the review highlights key implications for orthopedic practice, emphasizing early recognition, appropriate diagnostic strategies, and the importance of multidisciplinary management.

## 2. Methods

This narrative review was conducted in accordance with the Scale for the Assessment of Narrative Review Articles (SANRA) recommendations to ensure transparency, methodological rigor, and reproducibility. A structured literature search was performed across multiple electronic databases, including PubMed/MEDLINE, Embase, and Scopus, to identify relevant studies published from January 2000 to the present. This time frame was selected to capture contemporary classifications, diagnostic techniques, and advances in the biological understanding of T-cell lymphomas.

The search strategy combined controlled vocabulary and free-text terms related to T-cell lymphomas and musculoskeletal involvement. Core search terms included “T-cell lymphoma,” “peripheral T-cell lymphoma,” “anaplastic large cell lymphoma,” “NK/T-cell lymphoma,” and “adult T-cell leukemia/lymphoma,” in combination with “musculoskeletal,” “bone,” “osseous,” “skeletal,” “spine,” “muscle,” “soft tissue,” and “extranodal.” Additional targeted searches were performed to capture studies focusing on imaging characteristics, histopathology, immunophenotype, tumor microenvironment, and management considerations. Reference lists of relevant articles were manually screened to identify additional pertinent studies.

Eligible studies included original research articles, case series, case reports, and narrative or systematic reviews that described T-cell or NK/T-cell lymphomas with involvement of bone, bone marrow, skeletal muscle, joints, or soft tissues. Studies focusing exclusively on nodal or cutaneous disease without musculoskeletal extension were excluded. Only studies involving human subjects and published in English were considered.

Study selection was performed through an iterative screening process based on titles, abstracts, and full-text review. Given the rarity and heterogeneity of musculoskeletal manifestations of T-cell lymphomas, evidence was not restricted by study design. Included studies were grouped and synthesized according to major thematic domains aligned with the objectives of this review, including clinical and musculoskeletal manifestations, imaging features, pathology and immunophenotype, tumor microenvironment, and management considerations.

Data extraction was conducted qualitatively, with emphasis on identifying recurrent clinical patterns, diagnostic challenges, imaging hallmarks, and subtype-specific pathological and biological characteristics. Particular attention was given to studies providing detailed descriptions of musculoskeletal involvement and those offering insights into underlying biological mechanisms and tumor microenvironment interactions. The findings were narratively synthesized to provide a comprehensive and clinically relevant overview of this uncommon but important manifestation of T-cell lymphomas.

## 3. Clinical and Musculoskeletal Manifestations

### 3.1. Patterns of Bone Involvement

Bone involvement by lymphoma—whether primary or secondary—may present as unifocal or multifocal disease, a distinction with important prognostic implications. In a retrospective cohort of 127 patients with bone lymphoma, unifocal primary bone lymphoma most frequently involved the femur (32%), whereas multifocal disease showed a predilection for the spine and pelvis; importantly, multifocality was identified as an independent adverse prognostic factor for both progression-free and overall survival [8]. Clinically, patients typically present with localized bone pain—the most common symptom, reported in 76% of cases—often accompanied by soft tissue swelling (29%), neurological deficits (17%), or pathological fractures (16%). Axial skeletal involvement, particularly of the spine and pelvis (61%), may lead to spinal cord compression, representing a potentially urgent presentation. Systemic features such as fever, night sweats, and weight loss are less common but may coexist, while hypercalcemia—particularly associated with T-cell subtypes such as ATLL—can further obscure the clinical picture and lead to initial misattribution to infectious or degenerative orthopedic conditions [9].

#### Primary Versus Secondary Bone Involvement

An important clinical and staging distinction exists between primary bone lymphoma (PBL) and secondary osseous involvement in the context of systemic disease, as these entities differ in their diagnostic criteria, staging implications, and management approach. PBL is defined as lymphoma arising in bone without evidence of concurrent nodal or visceral disease at the time of diagnosis, corresponding to Ann Arbor Stage IE (unifocal osseous disease) or Stage IIE (regional nodal involvement without distant dissemination). By contrast, secondary bone involvement refers to osseous infiltration occurring as part of disseminated systemic lymphoma, typically classified as Stage IV disease. This distinction is clinically meaningful: PBL generally carries a more favorable prognosis and may be amenable to combined modality therapy with chemotherapy and consolidative radiotherapy, whereas secondary osseous involvement reflects advanced systemic disease requiring aggressive systemic treatment as the primary intervention. In T-cell lymphomas specifically, true PBL is exceedingly rare, with most reported cases representing secondary osseous involvement in the context of disseminated disease; this further underscores the importance of comprehensive staging with FDG-PET/CT at the time of diagnosis to accurately classify disease extent and guide treatment planning [9,10].

### 3.2. Soft-Tissue and Skeletal-Muscle Involvement

Soft tissue and skeletal muscle involvement by lymphoma is rare but diagnostically challenging, as it frequently mimics more common orthopedic and inflammatory conditions. In a retrospective MRI-based study of 20 patients with biopsy-proven skeletal muscle lymphoma, the predominant presentation was a muscle mass in 75% of cases, with diffuse homogeneous enhancement and fascial thickening observed in 84%, findings that overlap substantially with high-grade soft tissue sarcoma, inflammatory myositis, and infectious myonecrosis [11]. T-cell lymphomas are well represented in this setting. ALCL may arise primarily within skeletal muscle—most commonly in the lower extremities—presenting as a rapidly enlarging mass in younger patients; in one case, ALK-positive ALCL of the thigh was initially misclassified as a poorly differentiated malignancy on fine needle aspiration, with definitive diagnosis only achieved after core biopsy with CD30 and ALK immunostaining and confirmation of the t (2;5) translocation [12]. Similarly, extranodal NK/T-cell lymphoma has been reported to present as diffuse skeletal muscle involvement mimicking inflammatory myositis, with diagnosis ultimately established through biopsy demonstrating EBV-positive atypical lymphoid infiltrates [13].

### 3.3. Subtype-Specific Clinical Features

T-cell lymphoma subtypes exhibit distinct yet overlapping patterns of musculoskeletal involvement, reflecting differences in biology, disease distribution, and clinical behavior. Among these, anaplastic large-cell lymphoma (ALCL) demonstrates a notable predilection for osseous disease, particularly in younger patients, often presenting with osteolytic lesions that may be multifocal and preferentially involve the axial skeleton; in a clinicopathologic series, 50% of cases were multifocal and 83% involved axial sites, with a mean patient age of 33 years and a 5-year overall survival below 43%, indicating that osseous involvement portends an unfavorable prognosis irrespective of ALK status [14].

In contrast, peripheral T-cell lymphoma, not otherwise specified (PTCL-NOS), is characterized by marked heterogeneity and typically presents at advanced stages (III–IV), frequently with widespread extranodal disease. While bone involvement is less specifically defined than in ALCL, PTCL-NOS commonly affects the bone marrow, skin, liver, and gastrointestinal tract, and increasing extranodal burden—particularly multiorgan involvement—has been shown to independently predict poorer outcomes [15].

Extranodal NK/T-cell lymphoma (ENKTCL) demonstrates a distinct anatomical pattern, being classified into nasal and extranasal forms. While the nasal subtype predominates and typically presents at localized stages, extranasal disease—often involving the skin, gastrointestinal tract, testis, and occasionally skeletal muscle or bone marrow—tends to occur at more advanced stages and is associated with a worse prognosis. Importantly, musculoskeletal involvement in ENKTCL may manifest as diffuse FDG-avid skeletal muscle infiltration, closely mimicking inflammatory myopathies and further complicating early diagnosis [13].

In contrast to these patterns, adult T-cell leukemia/lymphoma (ATLL) is distinguished by its unique skeletal manifestations, particularly osteolysis and hypercalcemia. These features arise from tumor-mediated osteoclast activation driven by factors such as parathyroid hormone-related protein (PTHrP) and macrophage inflammatory protein-1α (MIP-1α), with experimental evidence demonstrating that PTHrP overexpression alone can induce significant bone resorption and trabecular loss. Clinically, this translates into a high prevalence of hypercalcemia—reported in up to 80% of patients with acute-type ATLL—representing a key diagnostic clue in the appropriate clinical context [16]. Key subtype-specific clinical and musculoskeletal features are summarized in Table 1.

### 3.4. Differential Diagnosis and Diagnostic Pitfalls

Musculoskeletal T-cell lymphoma presents a significant diagnostic challenge due to its close clinical and radiological resemblance to a wide range of orthopedic and oncologic conditions. In osseous disease, permeative or ’moth-eaten’ patterns of bone destruction on radiography or CT are indistinguishable from those seen in Ewing sarcoma—which further shares a predilection for younger patients and may demonstrate the classic onion-skin periosteal reaction and diaphyseal location—as well as atypical osteomyelitis, chronic osteomyelitis, multiple myeloma, and metastatic disease. Paradoxically, the relative preservation of cortical bone—more typical of lymphoma—may lead clinicians to underestimate disease aggressiveness and misdiagnose it as subacute infection, as illustrated in a case series where primary bone lymphoma was treated as osteomyelitis for several months prior to correct diagnosis [17].

**Figure 1:**
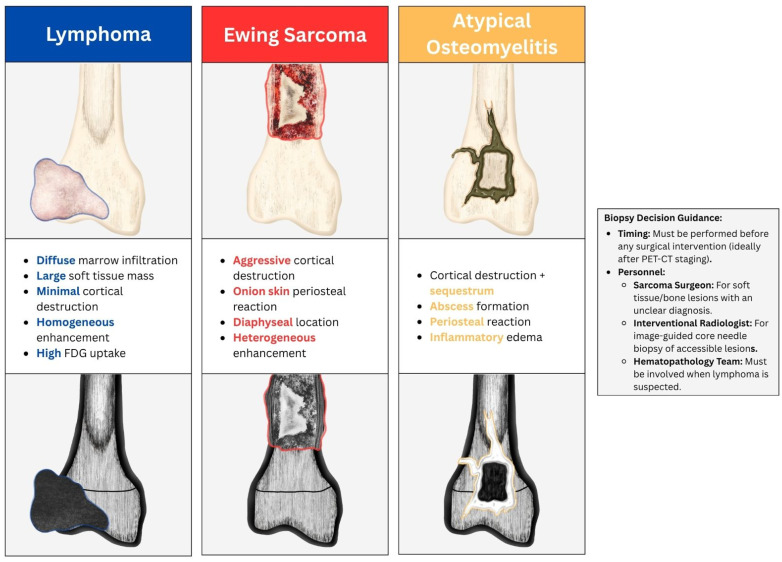
Comparative anatomic and radiographic features of musculoskeletal lymphoma, Ewing sarcoma, and atypical osteomyelitis, illustrating key distinguishing characteristics and biopsy decision guidance.

In soft tissue and muscle involvement, imaging features such as T2 hyperintensity, homogeneous enhancement, fascial thickening, and skin edema are non-specific and overlap with high-grade sarcoma, infectious myonecrosis, and inflammatory myopathies. A structured case series of seven biopsy-proven musculoskeletal lymphoma cases demonstrated that no single imaging modality was sufficient for definitive diagnosis; instead, accurate identification required multimodal imaging—including radiography, CT, MRI, and FDG-PET/CT—combined with tissue biopsy and immunohistochemical analysis [18]. Key distinguishing features between musculoskeletal T-cell lymphoma and its common mimics are summarized in Table 2. A comparative illustration of the distinguishing anatomic and radiographic features of musculoskeletal lymphoma, Ewing sarcoma, and atypical osteomyelitis—the two most clinically relevant and diagnostically challenging mimics—is presented in Figure 1, along with a practical biopsy decision guidance summary.

#### Differential Diagnosis with Other Lymphoma Subtypes Involving Bone

Beyond differentiation from non-lymphomatous conditions, musculoskeletal T-cell lymphoma must also be distinguished from other lymphoma subtypes that may involve bone, particularly diffuse large B-cell lymphoma (DLBCL) and Hodgkin lymphoma, as these entities share overlapping radiologic features but differ substantially in their histopathological characteristics, immunophenotype, and clinical behavior. DLBCL represents the most common cause of primary bone lymphoma, accounting for the majority of cases in most series, and typically presents with lytic osseous lesions and a large soft tissue component—features that are radiologically indistinguishable from T-cell lymphoma involvement. Hodgkin lymphoma, while less commonly presenting with primary bone involvement, may produce mixed lytic–sclerotic lesions and demonstrates a predilection for the axial skeleton, particularly the spine and pelvis [19]. The key to differentiation lies in tissue-based analysis: DLBCL demonstrates a B-cell immunophenotype with CD20, CD19, and PAX5 positivity and absence of T-cell markers, whereas Hodgkin lymphoma is characterized by the pathognomonic Reed–Sternberg cells expressing CD30 and CD15 with variable PAX5 positivity and a background reactive inflammatory infiltrate [20]. T-cell lymphomas, by contrast, express pan-T-cell markers such as CD3, CD2, and CD5, with subtype-specific markers including CD30 and ALK in ALCL, CD56 and EBER positivity in ENKTCL, and CD4/CD25/CCR4 in ATLL, as detailed in Section 5.2. Clinically, the relative rarity of T-cell bone lymphoma compared with B-cell disease—and the inferior prognosis associated with T-cell histology—make accurate immunophenotypic classification an essential component of the diagnostic workup in all cases of bone lymphoma [21]. Key differentiating features across lymphoma subtypes involving bone are summarized in Table 3.

### 3.5. Role of the Orthopedic Surgeon

Orthopedic surgeons play a pivotal role in the early recognition of musculoskeletal lymphoma, as they are often the first point of contact for patients presenting with bone or soft tissue complaints. Several clinical red flags should prompt suspicion for lymphoma, including disproportionate night pain, extensive marrow signal abnormalities on MRI without significant cortical destruction, large soft tissue masses relative to osseous involvement, multifocal lesions, elevated lactate dehydrogenase levels, systemic B symptoms, and hypercalcemia—particularly in T-cell lymphoma subtypes [22]. Recognition of these features should trigger early multidisciplinary collaboration with hematology and radiology prior to any surgical intervention.

**Figure 2:**
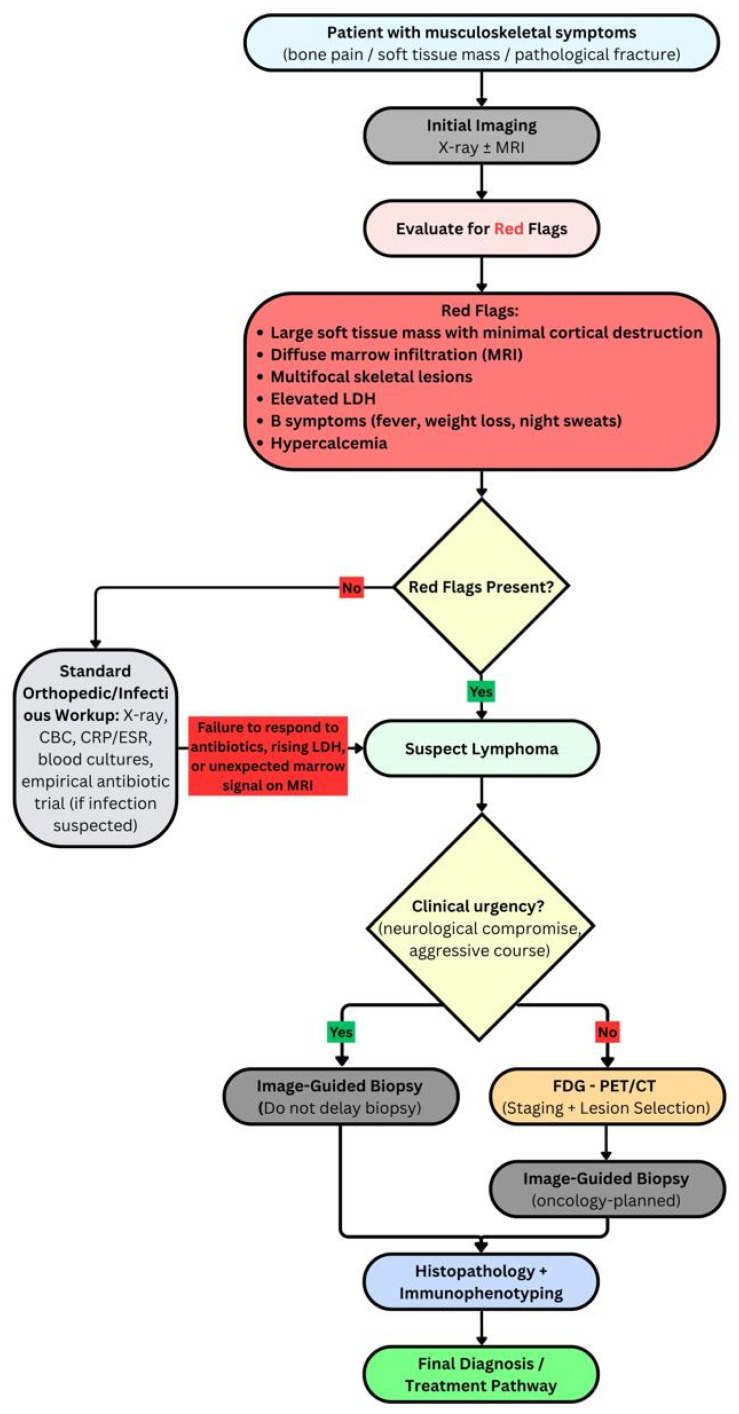
Diagnostic algorithm for suspected musculoskeletal lymphoma in orthopedic practice, integrating red flag recognition, standard orthopedic/infectious workup for low-suspicion cases, and a clinical urgency-stratified pathway leading to FDG-PET/CT-guided or immediate image-guided biopsy with histopathological confirmation.

The importance of appropriate biopsy planning cannot be overstated. In a reported case, a bone lesion initially suspected to represent high-grade chondrosarcoma based on imaging was ultimately diagnosed as diffuse large B-cell lymphoma following biopsy; proceeding directly to wide resection would have resulted in unnecessary morbidity and delayed systemic therapy [23]. Accordingly, all suspected malignant musculoskeletal lesions should undergo biopsy in an oncology-capable setting, with careful planning of the biopsy tract to allow future excision and with adequate tissue sampling for histopathology, immunohistochemistry, and flow cytometry to ensure accurate lymphoma subtyping. When red flags are absent, standard orthopedic and infectious workup should be initiated, including plain radiography, CBC, CRP/ESR, and blood cultures with empirical antibiotic therapy where infection is suspected; failure to respond to antibiotics, rising LDH, or an unexpected marrow signal on MRI should prompt re-evaluation for lymphoma. When red flags are present or clinical suspicion is raised, the need for FDG-PET/CT prior to biopsy should be weighed against clinical urgency: in stable patients, PET-CT is preferable to guide biopsy site selection toward the most metabolically active lesion, whereas neurological compromise or an aggressive disease course should prompt immediate image-guided biopsy without delay. A practical diagnostic algorithm integrating these decision points is summarized in Figure 2.

## 4. Imaging Features of Musculoskeletal T-Cell Lymphoma

### 4.1. Radiography and CT

On plain radiography and CT, primary bone lymphoma typically presents as either purely lytic (37%) or mixed lytic–sclerotic (49%) lesions with a permeative or “moth-eaten” pattern of bone destruction. A key diagnostic feature is the presence of a large extraosseous soft tissue component with relatively minimal cortical destruction—an imaging discordance observed in 13 of 15 large soft tissue masses in a 60-patient retrospective series. This pattern is atypical for aggressive primary bone tumors such as osteosarcoma or Ewing sarcoma, as well as for infectious processes, which more commonly produce pronounced cortical disruption. Recognition of this disproportion between extensive soft tissue extension and limited cortical involvement is therefore critical in raising suspicion for lymphoma in the pre-biopsy differential diagnosis [24].

### 4.2. Magnetic Resonance Imaging (MRI)

MRI provides superior characterization of disease extent and is essential for evaluating marrow and soft tissue involvement. Bone lymphoma lesions are typically infiltrative (91%), demonstrating diffuse hypointense marrow replacement on T1-weighted images and heterogeneous high signal intensity on T2-weighted sequences. In a dedicated MRI series of 29 patients, soft tissue extension was identified in 76% of cases, with the extraosseous component showing homogeneous T1 isointensity to muscle (90%), high T2 signal (91%), and predominantly homogeneous enhancement (82%). Cortical abnormalities—including permeative destruction, focal disruption, or cortical thickening—were present in 93% of cases, highlighting the ability of MRI to detect subtle cortical and marrow changes not fully appreciated on CT [25].

In cases of soft tissue or skeletal muscle lymphoma, MRI often demonstrates multicompartmental infiltration with diffuse T2 hyperintensity, fascial thickening, and homogeneous contrast enhancement, rather than a well-circumscribed mass. Although these findings overlap with inflammatory myopathies, the presence of diffuse involvement across muscle groups should raise suspicion for lymphoma, particularly when associated with marked FDG avidity on PET/CT [26].

### 4.3. FDG-PET/CT

FDG-PET/CT is the current standard for staging and response assessment in lymphoma, as established by the Lugano classification. Musculoskeletal lymphoma lesions typically exhibit intense and uniform 18F-FDG uptake, allowing for accurate detection of both skeletal and marrow involvement. In a prospective study of 66 pediatric lymphoma patients who underwent both FDG-PET/CT and bone marrow biopsy within a two-week interval, PET/CT demonstrated superior diagnostic performance, with an accuracy of 85%, specificity of 86%, and negative predictive value of 94%, compared to 59%, 53%, and lower predictive performance for bone marrow biopsy. Notably, PET/CT identified disease sites missed by biopsy, resulting in disease upstaging in a subset of patients [27]. Key imaging characteristics of musculoskeletal lymphoma across different modalities are summarized in Table 4.

## 5. Pathology, Immunophenotype, and Biological Basis

### 5.1. Biopsy Strategy for Musculoskeletal Lesions

Image-guided core needle biopsy (CNB) is the preferred initial diagnostic approach for suspected musculoskeletal lymphoma, achieving diagnostic success rates of 85–87% while enabling simultaneous acquisition of fresh tissue for ancillary studies, including flow cytometry (FCM), immunohistochemistry (IHC), fluorescence in situ hybridization (FISH), and T-cell receptor gene rearrangement analysis—techniques essential for accurate subclassification of T-cell lymphomas, which cannot be reliably diagnosed based on morphology alone. To preserve cellular viability for FCM, biopsy cores must be immediately placed in RPMI medium or saline rather than formalin, and sampling should target the most FDG-avid lesion on PET/CT rather than the most accessible site to avoid necrotic or fibrotic areas that may yield false-negative results [28].

Despite these advantages, several technical pitfalls may compromise diagnostic accuracy. Crush artifacts—resulting from excessive mechanical pressure during sampling—can distort cellular morphology and obscure immunophenotypic features, particularly in aggressive lymphoid malignancies. In addition, limited tissue volume, necrosis, and sclerosis may reduce cellular yield, leading to false-negative FCM results and difficulty distinguishing between PTCL-NOS, ALCL, and non-lymphomatous mimics. In bone lymphoma specifically, up to 30% of cases require repeat biopsy due to insufficient viable tissue or sampling error, emphasizing the importance of meticulous biopsy planning and coordination with experienced hematopathology teams [29].

### 5.2. Histology and Immunophenotype by Subtype

Musculoskeletal T-cell lymphomas share overlapping cytologic features but are distinguished by subtype-specific immunophenotypic profiles that are essential for accurate classification and for differentiating these entities from mimics such as Hodgkin lymphoma or sarcoma. Anaplastic large-cell lymphoma (ALCL) is characterized by large pleomorphic lymphoid cells with abundant cytoplasm and hallmark horseshoe- or kidney-shaped nuclei, demonstrating strong and uniform CD30 expression. ALK-positive cases exhibit nuclear–cytoplasmic ALK staining due to underlying ALK gene rearrangements, whereas ALK-negative cases lack ALK expression but retain the characteristic CD30-bright phenotype; importantly, CD45 positivity and absence of PAX5 help distinguish ALCL from classic Hodgkin lymphoma despite overlapping CD30 expression [30].

In contrast, peripheral T-cell lymphoma, not otherwise specified (PTCL-NOS), demonstrates marked morphologic and immunophenotypic heterogeneity, typically expressing pan-T-cell markers such as CD2, CD3, and CD5, with CD4 predominance and frequent loss of CD7. A substantial subset exhibits a T-follicular helper (TFH) phenotype, defined by expression of PD-1, CXCL13, ICOS, and BCL6, which overlaps with TFH lymphomas and necessitates extended immunophenotypic panels in diagnostically ambiguous cases [31].

Extranodal NK/T-cell lymphoma (ENKTCL) further contrasts with these entities by demonstrating a distinctive angiocentric and angiodestructive growth pattern with prominent necrosis. Tumor cells express cytoplasmic or surface CD3, CD2, and cytotoxic markers such as TIA-1 and granzyme B, frequently coexpress CD56, and are uniformly associated with Epstein–Barr virus infection, confirmed by EBER in situ hybridization—an essential diagnostic feature that distinguishes ENKTCL from other aggressive T-cell neoplasms with similar morphology [32].

**Figure 3:**
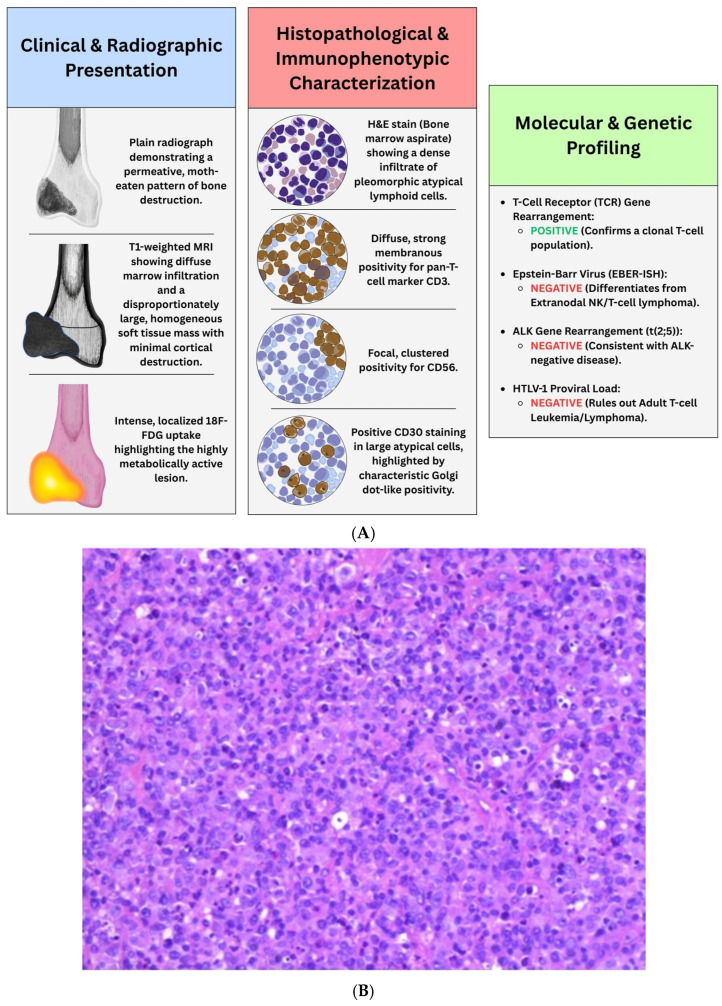
(**A**) Illustrative composite of musculoskeletal T-cell lymphoma. Integrated diagnostic framework depicting clinical and radiographic presentation, immunophenotypic characterization, and molecular profiling findings; (**B**) representative histopathological photomicrograph from a published case of primary bone peripheral T-cell lymphoma, demonstrating a dense infiltrate of small round atypical lymphoid cells arranged in solid sheets on H&E staining (adapted from Wang et al., Transl Cancer Res, 2016) [35].

Finally, adult T-cell leukemia/lymphoma (ATLL) is characterized by pleomorphic infiltrates of atypical lymphocytes with multilobated “flower-like” nuclei and a characteristic immunophenotype of CD4+, CD25+, CD3+, and CD5+, with frequent loss of CD7. More refined flow cytometric signatures—particularly CD4+ CCR4+ CD26− CD7− profiles—have demonstrated high specificity for ATLL and correlate with HTLV-1 proviral load, supporting diagnosis when integrated with serologic and molecular findings [33,34]. Key immunophenotypic features distinguishing T-cell lymphoma subtypes are summarized in Table 5. An illustrative composite case demonstrating the integrated clinical, radiographic, histopathological, and molecular findings of musculoskeletal T-cell lymphoma is presented in Figure 3.

### 5.3. Tumor Microenvironment in Bone and Soft Tissue

The tumor microenvironment (TME) of musculoskeletal T-cell lymphomas is a complex and dynamic ecosystem composed of stromal cells, immune infiltrates, osteoclasts and osteoblasts, endothelial elements, and a network of cytokines and chemokines that collectively regulate tumor growth, immune evasion, and therapeutic response [36,37]. In peripheral T-cell lymphomas, single-cell RNA sequencing of bone marrow involvement has revealed pronounced immune heterogeneity, including enrichment of exhausted cytotoxic T cells and NK cells, activated monocytes and neutrophils, and pro-inflammatory gene signatures—features that correlate with treatment response and adverse clinical outcomes [37].

**Figure 4:**
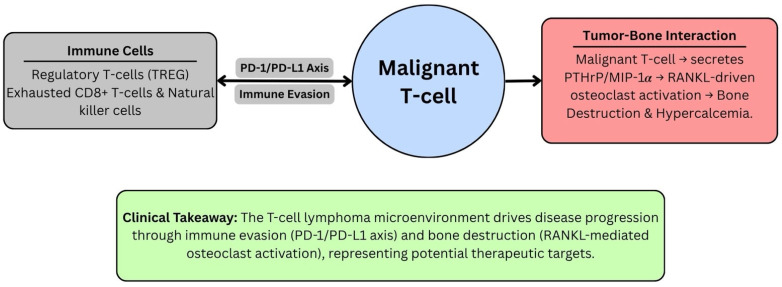
Key cellular and molecular interactions within the tumor microenvironment of musculoskeletal T-cell lymphoma, illustrating immune evasion through the PD-1/PD-L1 axis and RANKL-mediated osteoclast activation driving bone destruction and hypercalcemia, representing potential therapeutic targets.

Insights from cutaneous T-cell lymphoma further illustrate how malignant T cells exploit the surrounding microenvironment, adopting T helper 2 (Th2)-skewed programs and interacting with regulatory T cells, B-cell aggregates resembling tertiary lymphoid structures, and antigen-presenting stromal cells to sustain tumor survival [36]. Within the bone microenvironment, neoplastic T cells—particularly in ATLL—secrete osteoclast-activating factors such as parathyroid hormone-related protein (PTHrP) and macrophage inflammatory protein-1α, driving osteolysis through RANKL-dependent pathways and associated cytokine signaling, including IL-6. These interactions not only promote bone destruction but also create a permissive niche that facilitates tumor growth, migration, and survival [16]. The principal cellular and molecular interactions within the tumor microenvironment of musculoskeletal T-cell lymphomas are illustrated in Figure 4.

These biological insights have important translational implications. The presence of immune exhaustion and PD-1/PD-L1 signaling within the TME has been successfully targeted in extranodal NK/T-cell lymphoma, where PD-1 blockade with pembrolizumab induced responses in all seven heavily pretreated patients, including five complete remissions, with manageable toxicity [32,38]. Such findings support the integration of immune checkpoint inhibition with therapies targeting stromal and bone-related pathways—such as RANKL or IL-6—as a rational strategy for TME-directed treatment in T-cell lymphomas.

## 6. Management and Outcomes

Systemic therapy remains the cornerstone of treatment for musculoskeletal T-cell lymphomas, with regimen selection guided by histologic subtype. For most peripheral T-cell lymphoma (PTCL) subtypes, anthracycline-based combination chemotherapy remains standard, with CHOP (cyclophosphamide, doxorubicin, vincristine, prednisone) or CHOEP commonly used as first-line regimens. Population-based data involving 906 patients demonstrated that CHOEP provides superior outcomes compared with CHOP in eligible patients, with improved 5-year progression-free survival (32.9% vs. 25.0%) and overall survival (65.6% vs. 47.6%) [39]. In CD30-positive PTCL, particularly systemic anaplastic large-cell lymphoma (sALCL), the therapeutic landscape has evolved significantly following the ECHELON-2 phase III trial, which demonstrated that brentuximab vedotin plus CHP (A+CHP) reduced the risk of progression or death by 29% and the risk of death by 34% compared with CHOP, with higher complete remission rates (71% vs. 53%) and sustained survival benefits at 5-year follow-up, establishing A+CHP as the current standard of care in this setting [40].

For extranodal NK/T-cell lymphoma (ENKTCL), asparaginase-based regimens constitute the backbone of therapy. A randomized trial comparing DDGP (cisplatin, dexamethasone, gemcitabine, pegaspargase) with SMILE in advanced-stage disease demonstrated significantly improved outcomes with DDGP, including higher overall response rates (90.0% vs. 60.0%), superior 3-year progression-free survival (56.6% vs. 41.8%), and improved 5-year overall survival (74.3% vs. 51.7%), supporting its use as a preferred regimen in high-burden disease [41]. In adult T-cell leukemia/lymphoma (ATLL), outcomes remain poor, and treatment options are limited; however, targeted therapy with the CCR4 monoclonal antibody mogamulizumab has emerged as an important salvage option. In a phase II study of relapsed or refractory ATLL, mogamulizumab demonstrated superior response rates and progression-free survival compared with conventional chemotherapy, although median overall survival remained approximately 14 months, underscoring the aggressive nature of this disease [42].

Radiotherapy (RT) plays a selective but important role in the management of localized musculoskeletal lymphoma. In a large institutional study of 1948 patients, consolidative RT to skeletal sites (typically 30–40 Gy) was independently associated with improved local control and overall survival in patients achieving a response to chemotherapy, particularly in aggressive subtypes and those with residual PET-positive disease [43]. In primary bone lymphoma, combined modality therapy with chemotherapy and RT has traditionally been standard; however, contemporary evidence suggests that RT may be omitted in patients achieving complete metabolic remission, with its use increasingly reserved for partial responders or those with residual disease [44].

**Figure 5:**
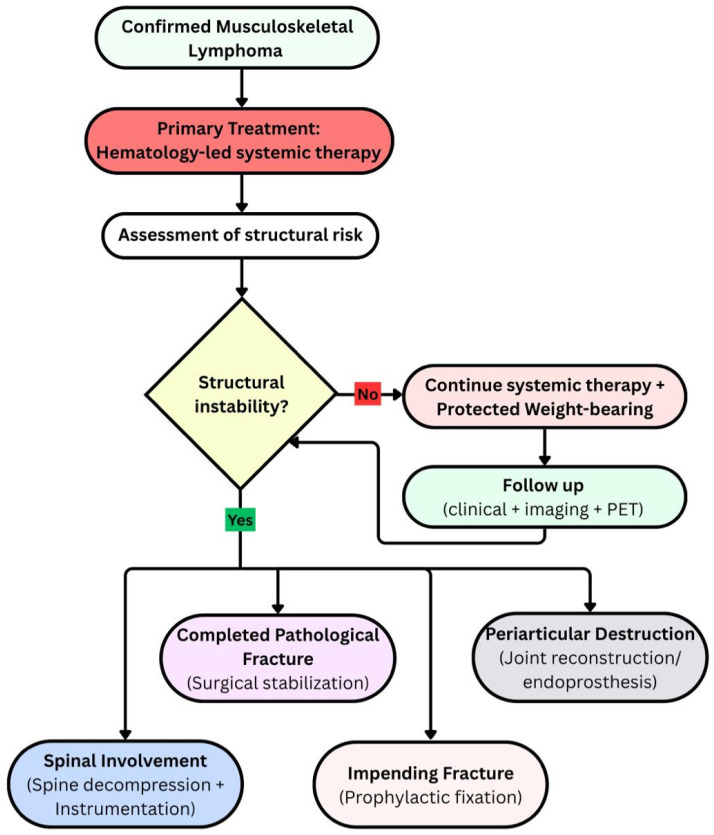
Orthopedic management pathway for confirmed musculoskeletal lymphoma, illustrating structural risk-stratified surgical decision-making and the indications for intervention versus continuation of systemic therapy with surveillance.

From an orthopedic perspective, biopsy remains the critical first step and must be carefully planned to avoid contamination of future surgical planes. Once lymphoma is confirmed, systemic therapy—with or without RT—takes priority, and surgical intervention is generally reserved for structural complications. Indications for orthopedic management include prophylactic fixation of impending fractures in weight-bearing bones with significant cortical destruction, stabilization of completed pathological fractures, spinal decompression and instrumentation for neurologic compromise, and joint reconstruction in cases of periarticular destruction [38]. Peri-treatment considerations include minimizing tumor spillage during biopsy, maintaining protected weight-bearing during therapy, and coordinating surgical timing with hematologic recovery. Once lymphoma is confirmed, systemic therapy takes priority; orthopedic intervention is reserved for cases of structural instability, with the specific surgical approach guided by the nature of skeletal compromise—including prophylactic fixation for impending fractures, stabilization of completed pathological fractures, spinal decompression and instrumentation for neurological compromise, and joint reconstruction for periarticular destruction. Structurally stable patients should continue systemic therapy under protected weight-bearing with clinical, imaging, and PET-based surveillance. This decision framework is illustrated in Figure 5.

Outcome data specific to T-cell primary bone lymphoma (T-PBL) remain limited but consistently demonstrate inferior prognosis compared with B-cell counterparts. In a retrospective cohort of 61 patients, T-cell lymphoma accounted for 18% of cases, with the overall 5-year survival of 52.3% largely driven by diffuse large B-cell lymphoma, while T-cell cases exhibited poorer responses and earlier metastatic progression [21]. Individual case reports further illustrate the aggressive clinical course, with rapid disease progression, early distant metastasis, and death within months despite combined modality treatment, highlighting the unfavorable biology of T-cell PBL relative to B-cell disease [45]. Key orthopedic considerations in the management of musculoskeletal lymphoma are summarized in Table 6.

## 7. Knowledge Gaps and Future Directions

Despite growing recognition of musculoskeletal manifestations in T-cell lymphomas, several critical knowledge gaps persist that are specific to this rare clinical entity. First, prospective multicenter data on T-cell primary bone lymphoma (T-PBL) remain virtually absent. Even large population-based analyses are overwhelmingly dominated by B-cell histologies, with T- and NK-cell subtypes comprising fewer than 5% of cases, rendering subtype-specific conclusions about prognosis, recurrence patterns, and treatment outcomes largely inferential [10]. Establishing dedicated registries for T-PBL and related osseous T-cell entities represents an urgent and achievable priority.

From a diagnostic standpoint, the radiologic overlap between musculoskeletal T-cell lymphoma and its closest mimics—particularly Ewing sarcoma and atypical osteomyelitis—continues to cause diagnostic delays with meaningful prognostic consequences. MRI-based radiomic models and machine learning algorithms have demonstrated promising performance in distinguishing malignant from benign bone lesions; however, these tools have been developed almost exclusively on sarcoma and metastatic datasets, with no dedicated models validated for lymphoma versus its musculoskeletal mimics [46]. The development and external validation of such tools represent a high-impact and feasible research priority given the routine use of MRI in this setting.

Regarding biopsy strategy, optimal protocols for musculoskeletal T-cell lymphoma remain incompletely defined. Key unresolved questions include the minimum tissue volume required for reliable flow cytometry and T-cell receptor gene rearrangement analysis in bone lesions, the optimal number of core passes to minimize false-negative rates—currently estimated at up to 30% requiring repeat biopsy—and the threshold for escalation to open biopsy when image-guided sampling is non-diagnostic [28,29]. Prospective studies addressing these technical parameters in the specific context of suspected bone lymphoma are needed.

Therapeutically, the role of consolidative radiotherapy in musculoskeletal T-cell lymphoma achieving complete metabolic remission on PET/CT remains undefined, with current practice extrapolated from B-cell primary bone lymphoma data. Similarly, the threshold for prophylactic surgical fixation in weight-bearing bones during systemic therapy—including the influence of treatment response on fracture risk—has not been studied in T-cell histologies specifically, and evidence-based perioperative guidelines for this population are lacking [38,43,44].

Finally, the translational implications of the bone microenvironment in T-cell lymphoma warrant focused investigation. The RANKL-PTHrP axis driving osteolysis in ATLL and the immune exhaustion phenotype observed in bone marrow involvement by PTCL represent rational therapeutic targets; however, clinical data on the combination of checkpoint inhibition, anti-resorptive agents, or epigenetic therapies in the musculoskeletal context remain absent [16,47]. Addressing these gaps through integrated multidisciplinary and translational research will be essential to improving outcomes in this rare but challenging disease spectrum.

## 8. Conclusions

Musculoskeletal involvement in T-cell lymphomas represents a rare but clinically significant and under-recognized manifestation that poses substantial diagnostic and therapeutic challenges. Owing to its ability to mimic common orthopedic and infectious conditions, early recognition requires a high index of suspicion and careful integration of clinical presentation with imaging findings. In this context, radiologic features—particularly the combination of diffuse marrow infiltration, soft tissue extension, and disproportionate cortical preservation—serve as critical clues that should prompt timely biopsy and further hematologic evaluation. Definitive diagnosis, however, relies on comprehensive pathologic assessment, including immunophenotyping and molecular studies, which remain indispensable for accurate subtype classification and appropriate treatment selection.

Beyond diagnosis, increasing understanding of the biological and tumor microenvironmental landscape of T-cell lymphomas offers important insights into disease behavior and therapeutic resistance, highlighting the potential for targeted and TME-directed strategies in future management.

This narrative review synthesizes current evidence across clinical, radiologic, and biologic domains, providing a multidisciplinary perspective that is particularly relevant to orthopedic practice. Its strengths include a comprehensive, integrative approach to a rare clinical entity and emphasis on practical diagnostic and management considerations. However, it is limited by the inherent constraints of a narrative design and the paucity of high-quality, subtype-specific data, which remain largely confined to retrospective studies and case reports. Continued multicenter collaboration and translational research will be essential to advance understanding and improve outcomes in this challenging disease spectrum.

## Figures and Tables

**Table 1:** Subtype-specific musculoskeletal features of T-cell lymphomas.

Subtype	Typical Age Group	Musculoskeletal Involvement Pattern	Key Clinical Features	Biological/Pathophysiological Features	Prognosis
**ALCL (ALK±)**	Younger patients (mean ~33 years)	Frequent bone involvement; often osteolytic; axial skeleton predominance; may be multifocal	Bone pain, swelling, possible pathological fractures	CD30+; ALK rearrangements (ALK+ subtype); aggressive tumor behavior in bone	Poor when involving bone (<43% 5-year OS)
**PTCL-NOS**	Middle-aged to older adults	Widespread extranodal disease; bone marrow involvement common; less specific bone predilection	Advanced-stage disease; systemic symptoms; multiorgan involvement	Highly heterogeneous; variable immunophenotype; T-follicular helper subsets	Poor, worsened by extranodal burden
**ENKTCL**	Variable (more common in Asian populations)	Primarily nasal; extranasal disease may involve muscle, soft tissue, marrow	Destructive midline lesions (nasal); muscle involvement mimicking myositis	EBV-driven; CD56+; cytotoxic phenotype; angiocentric growth	Worse in extranasal disease
**ATLL**	Adults in endemic regions	Osteolysis; marrow infiltration; diffuse skeletal involvement	Hypercalcemia (very common), bone pain, systemic disease	HTLV-1 associated; PTHrP and MIP-1α driven osteoclast activation	Aggressive, poor prognosis

**Abbreviations:** ALCL, anaplastic large-cell lymphoma; PTCL-NOS, peripheral T-cell lymphoma, not otherwise specified; ENKTCL, extranodal NK/T-cell lymphoma; ATLL, adult T-cell leukemia/lymphoma; OS, overall survival; ALK, anaplastic lymphoma kinase; EBV, Epstein–Barr virus; HTLV-1, human T-lymphotropic virus type 1; PTHrP, parathyroid hormone-related protein.

**Table 2:** Differential diagnosis of musculoskeletal T-cell lymphoma.

Feature	T-Cell Lymphoma	Osteomyelitis	Ewing Sarcoma	Osteosarcoma	Multiple Myeloma	Metastasis
**Age**	Variable (often adults; ALCL younger)	Any	Children/young adults	Adolescents	Older adults	Older adults
**Pain**	Persistent, progressive, often night pain	Acute/subacute, may improve with antibiotics	Progressive	Progressive	Chronic	Variable
**Systemic symptoms**	B symptoms, hypercalcemia (ATLL), ↑LDH	Fever, infection signs	Possible fever	Rare	Anemia, fatigue	Depends on primary
**Bone pattern**	Permeative/lytic ± minimal cortical destruction	Lytic with sequestrum	Permeative	Mixed lytic/blastic	Lytic (“punched out”)	Lytic/blastic
**Soft tissue mass**	Large, disproportionate to bone destruction	Variable	Common	Common	Rare	Variable
**MRI pattern**	Diffuse marrow infiltration	Localized inflammation	Aggressive mass	Tumor matrix	Marrow replacement	Variable
**PET/CT**	High FDG uptake	Moderate uptake	High	High	Variable	Variable
**Key clue**	Minimal cortical destruction + large soft tissue mass	Infection labs	Age + diaphyseal lesion	Bone formation	Multiple lesions	Known primary

**Table 3:** Differential diagnosis of lymphoma subtypes involving bone.

Feature	T-Cell Lymphoma	DLBCL (B-Cell)	Hodgkin Lymphoma
**Frequency of bone involvement**	Rare (~5% of bone lymphoma)	Most common (majority of PBL cases)	Uncommon; secondary > primary
**Typical age group**	Variable; subtype-dependent	Middle-aged to older adults	Young adults (bimodal)
**Imaging pattern**	Permeative lytic; large soft tissue mass; minimal cortical destruction	Lytic; large soft tissue mass	Mixed lytic–sclerotic; axial predilection
**Key histological features**	Pleomorphic atypical T-lymphocytes; subtype-specific morphology	Large B-cells with vesicular nuclei	Reed–Sternberg cells; reactive background
**Immunophenotype**	CD3+, CD2+, CD5+; subtype markers (CD30, ALK, CD56, EBER)	CD20+, CD19+, PAX5+; T-cell markers negative	CD30+, CD15+, PAX5 (weak/variable); CD20−
**EBV association**	ENKTCL subtype (EBER+)	Subset (~10–15%)	Mixed cellularity subtype
**Prognosis**	Poor; inferior to B-cell disease	Intermediate; favorable with R-CHOP	Generally favorable with combined modality therapy
**First-line treatment**	Subtype-specific (CHOP/CHOEP, A+CHP, asparaginase-based)	R-CHOP ± radiotherapy	ABVD or BEACOPP ± radiotherapy

**Table 4:** Imaging hallmarks of musculoskeletal lymphoma.

Modality	Key Findings	Diagnostic Clues
**Radiography/CT**	Lytic or mixed lesions; permeative/moth-eaten pattern	Disproportionately large soft tissue mass with minimal cortical destruction
**MRI**	Diffuse marrow replacement (T1 low, T2 high); homogeneous enhancement	Multicompartment involvement; neurovascular encasement
**Soft tissue MRI**	Diffuse muscle infiltration; fascial thickening	Lack of well-defined mass → favors lymphoma over sarcoma
**FDG-PET/CT**	Intense FDG uptake; detects multifocal disease	Superior to bone marrow biopsy; guides biopsy site
**Comparative clue**	Lymphoma vs. sarcoma	Less cortical destruction, more diffuse infiltration

**Table 5:** Immunophenotypic features of T-cell lymphoma subtypes. ↓, decreased/lost expression.

Subtype	Key Markers	Additional Features	Diagnostic Clues
**ALCL (ALK+)**	CD30+, ALK+, CD45+	NPM1–ALK fusion	Younger patients; better prognosis
**ALCL (ALK−)**	CD30+, ALK−	Molecular heterogeneity	Worse prognosis than ALK+
**PTCL-NOS**	CD2+, CD3+, CD5+, CD4+, ↓CD7	TFH markers (PD-1, CXCL13, ICOS, BCL6)	Highly heterogeneous
**ENKTCL**	CD3+, CD56+, TIA-1+, Granzyme B+	EBER+ (EBV-associated)	Angiocentric necrosis
**ATLL**	CD4+, CD25+, CCR4+, CD26−, CD7−	HTLV-1 associated	Hypercalcemia + “flower cells”

**Table 6:** Orthopedic considerations in musculoskeletal lymphoma.

Scenario	Indication	Intervention
Suspected malignant lesion	Diagnosis	Image-guided biopsy (oncology-planned)
Impending fracture	>50% cortical destruction	Prophylactic fixation
Pathologic fracture	Structural failure	Surgical stabilization
Spinal involvement	Neurological compromise	Decompression + instrumentation
Periarticular destruction	Joint collapse risk	Reconstruction (e.g., endoprosthesis)
During chemo	Weak bone	Protected weight-bearing
Key principle	Always	Treat lymphoma first (systemic therapy before major surgery)

## Data Availability

The original contributions presented in this study are included in the article. Further inquiries can be directed to the corresponding author.
